# The chromatin architectural proteins HMGD1 and H1 bind reciprocally and have opposite effects on chromatin structure and gene regulation

**DOI:** 10.1186/1471-2164-15-92

**Published:** 2014-02-01

**Authors:** Narasimharao Nalabothula, Graham McVicker, John Maiorano, Rebecca Martin, Jonathan K Pritchard, Yvonne N Fondufe-Mittendorf

**Affiliations:** 1 Department of Molecular and Cellular Biochemistry, University of Kentucky, Lexington, KY 40536, USA; 2 Department of Biostatistics and Computational Biology, Dana-Farber Cancer Institute and Harvard School of Public Health, Boston, MA 02115, USA; 3 Department of Genetics, Stanford University, Stanford, CA 94305, USA; 4 Department of Molecular Biosciences, Northwestern University, Evanston, IL 60208, USA; 5 Department of Biology, Stanford University, Stanford, CA 94305, USA; 6 Howard Hughes Medical Institute, Stanford University, Stanford, CA 94305, USA

**Keywords:** Chromatin structure, Transcriptional regulation, Histone H1, High mobility group protein, Nucleosome repeat length

## Abstract

**Background:**

Chromatin architectural proteins interact with nucleosomes to modulate chromatin accessibility and higher-order chromatin structure. While these proteins are almost certainly important for gene regulation they have been studied far less than the core histone proteins.

**Results:**

Here we describe the genomic distributions and functional roles of two chromatin architectural proteins: histone H1 and the high mobility group protein HMGD1 in *Drosophila* S2 cells. Using ChIP-seq, biochemical and gene specific approaches, we find that HMGD1 binds to highly accessible regulatory chromatin and active promoters. In contrast, H1 is primarily associated with heterochromatic regions marked with repressive histone marks. We find that the ratio of HMGD1 to H1 binding is a better predictor of gene activity than either protein by itself, which suggests that reciprocal binding between these proteins is important for gene regulation. Using knockdown experiments, we show that HMGD1 and H1 affect the occupancy of the other protein, change nucleosome repeat length and modulate gene expression.

**Conclusion:**

Collectively, our data suggest that dynamic and mutually exclusive binding of H1 and HMGD1 to nucleosomes and their linker sequences may control the fluid chromatin structure that is required for transcriptional regulation. This study provides a framework to further study the interplay between chromatin architectural proteins and epigenetics in gene regulation.

## Background

Eukaryotic DNA is packaged into chromatin, a highly compacted structure made up of repeating nucleosome units. Nucleosomes consist of 147 bp of DNA wrapped around an octamer of core histones (two copies each of H2A, H2B, H3 and H4) and are connected to each other by short stretches of linker DNA [1,2]. In most organisms, this short extra-nucleosomal linker region is bound by an additional histone, known as H1. H1 belongs to a class of chromatin architectural proteins (CAPs) that are responsible for maintaining, modulating and stabilizing chromatin architecture. H1 binds to the DNA as it enters/exits the nucleosome and may compact chromatin into a higher-order fiber structure [3-7]. This is supported by *in vivo* studies that have shown that reducing the total amount of cellular H1 results in a less compact chromatin structure [8-11].

The ability of H1 to compact chromatin may be antagonized by other CAPs, such as the highly abundant high mobility group proteins (HMGs). HMGs decompact higher-order chromatin structures to promote the binding of nuclear regulatory factors to their binding sites [12-17]. HMGs have similar DNA and chromatin binding properties to H1 [18-22], bind to sites at the entry/exit dyad of the nucleosome and linker DNA [23], and may out-compete H1 in order to activate specific transcriptional programs [16,17,19,24]. Furthermore, changes in the concentration of H1 and HMG proteins alter transcriptional programs important for normal cell development and viability [11,15,25-31]. Thus, H1 and HMG proteins are both chromatin architectural proteins that may serve as active regulators of transcription.

Similar chromatin binding of H1 and HMGs suggests that they may be functionally linked and act in opposition with respect to the stability of chromatin structure [28,30]. Potentially, access to the genome by transcription regulatory machinery could be mediated by competition between H1 and HMG. Support for this hypothesis comes from early embryogenesis, where initially HMGD1 (*Drosophila*’s only known HMG) or HMG-B *(Xenopus)* is highly abundant and H1 is barely detectable. As development progresses, H1 is expressed and replaces HMGD1/HMGB1 at some regions of the genome [19,32]. This replacement is thought to silence specific genes and thus contribute to programmed development.

Despite suggestive evidence of a relationship between HMGs and H1, their genome-wide distributions are not known and a clear understanding of how they are related to chromatin structure and gene expression is lacking. Here we apply genome-wide profiling and gene specific approaches to study these proteins in *D. melanogaster* S2 cells and to better understand their roles in gene regulation and chromatin structural changes. As *Drosophila* only encodes one isoform each of histone H1 and HMGD1, and there exists a wealth of other genomics data for this cell line, S2 cells are an excellent model system for studying this problem.

Here, we report detailed experiments and analyses that show that H1 and HMGD1 are associated with specific genomic regions with specific transcriptional activity. We show that these proteins bind reciprocally with each other and affect gene regulation and local chromatin structures. The data we have generated serves as a useful resource for understanding the interplay between histone modifications, chromatin architectural proteins, chromatin structure and gene expression.

## Results

### H1 and HMGD are enriched in different chromatin regions

**Figure 1:**
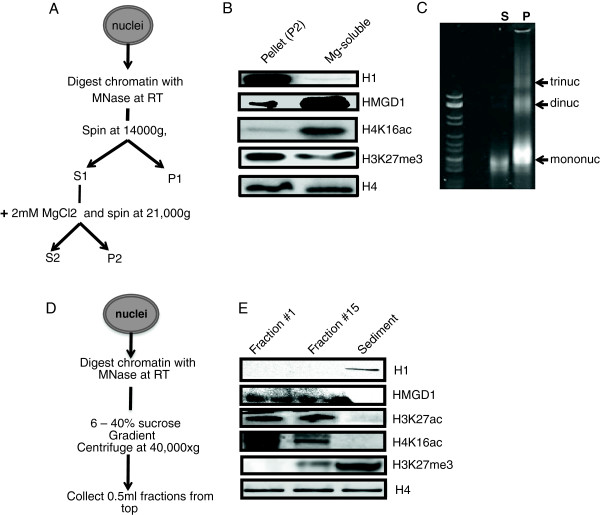
**HMGD and H1 are associated with euchromatic and heterochromatic fractions respectively. (A)** Salt fractionation of chromatin procedure. **(B)** Western blot of H1 and HMGD1 in heterochromatic (P2) and euchromatic (S2) chromatin fractions. We digested chromatin from S2 nuclei with micrococcal nuclease and fractionated it to yield supernatant (S2) and pellet (P2) fractions. The western blot shows that H1 is primarily found in the heterochromatic fraction (P2) and HMGD1 is primarily found in the euchromatic fraction (S2). The histone mark H4K16ac is a positive marker for active euchromatin and the histone H4 antibody is used as a chromatin loading marker. **(C)** Ethidium bromide stained 3.3% Nusieve™ agarose gel showing the DNA associated with the S2 (magnesium-soluble) and P (magnesium-insoluble) fractions. **(D)** Sucrose gradient fractionation procedure. **(E)** Western blot analysis of H1 and HMGD1 released from MNase-digested chromatin from S2 cells. The 6 – 40% sucrose gradient reveals that H1 is bound to the heavier heterochromatin, while HMGD1 is bound to the lighter euchromatin.

To test whether HMGD1 and H1 are associated with distinct chromatin states, we first determined the relative abundance of these proteins in different chromatin fractions. We isolated nuclei from *D. melanogaster* S2 cells and digested their chromatin with micrococcal nuclease (MNase). We subjected the solubilized chromatin to salt fractionation analyses (Figure 1A) and analyzed the resulting euchromatic (soluble) and heterochromatic (insoluble) fractions by agarose gel (Figure 1C) and western blot (Figure 1B). The majority of HMGD1 protein is associated with the active euchromatic fraction as typified by the presence of H4K16ac (active histone mark), while most of H1 is present in the heterochromatic fraction (Figure 1B). We confirmed these results using a slightly modified version of the sucrose gradient fractionation method [33] (Figure 1D & E). Here too, H1 sedimented with the highly dense, longer and inactive chromatin and HMGD1 associated with the lighter, shorter and active chromatin fractions. While both results show that HMGD1 and H1 associate preferentially with euchromatin and heterochromatin respectively, a non-negligible proportion of each protein is present in both fractions. This could be due to post translational modifications of these proteins, which may change their targets and cellular localization.

To gain further insight into the functional consequences of HMGD1 and Histone H1 binding to chromatin, we used nucleosome-ChIP-seq to obtain genome-wide maps of HMGD1- and H1 -bound nucleosomes in *D. melanogaster* S2 cells. We first digested chromatin with MNase to yield ~450 bp fragments, which are long enough to include linker DNA bound by either H1 or HMGD1. We then immunoprecipitated chromatin fragments using ChIP-grade antibodies against Histone H1, HMGD1, and IgG (Additional file 1: Figure S1) and adapted the immunoprecipitated mononucleosomal fragments for SoLiD sequencing. After sequencing the reads, we aligned them to the reference genome and discarded those that did not map uniquely. In total, we mapped 12–14 million unique reads, equivalent to ~18-22× fold coverage per nucleosome (Additional file 1: Table S1). Total nucleosomal input DNA from S2 cells was also sequenced and used to correct for background nucleosome occupancy.

**Figure 2:**
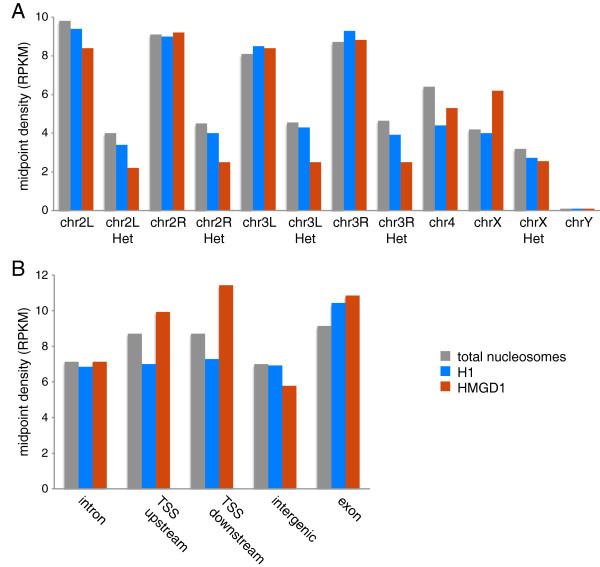
**Density of HMGD1 and H1 across different chromosomes and genomic regions in S2 cells. (A)** Density of midpoints from mapped HMGD1 and H1 ChIP-seq reads on each chromosome. **(B)** Density of HMGD1 and H1 ChIP-seq reads in different genomic regions: promoters (defined as 1 kb upstream and downstream of TSSs), exons, introns, and intergenic regions in the *D. melanogaster* genome. “Upstream” and “Downstream” regions are defined as 1 kb upstream and 1 kb downstream of the transcription start site, respectively.

We examined the broad distribution of H1– and HMGD1-bound nucleosomes across chromosomes. After normalizing the total number of mapped reads in the H1 and HMGD1 datasets, we found that HMGD1-bound nucleosomes are consistently depleted on the heterochromatic chromosome arms compared to both H1 and total nucleosomes (Figure 2A and Additional file 1: Table S1). Interestingly, HMGD1 is highly abundant on the X chromosome compared to total nucleosomes. Potentially this could be related to dosage compensation in male flies (S2 cells are biologically male), which results in a doubling of gene expression on the X-chromosome (Meller and Kuroda 2002). These results are consistent with those from the chromatin fractionation experiments and indicate that HMGD1 is enriched in the euchromatic fraction of the genome.

### HMGD1 and H1 presence at transcription start sites correlates with gene expression

The above results prompted us to ask whether the occupancy of HMGD1 and H1 vary across different chromosomal regions and features that are associated with transcription. We used the flybase gene annotations to define genomic regions as intergenic, intronic, or exonic, and we labeled regions within 1000 bp of annotated transcription start sites (TSS) as promoters. To estimate the relative abundance of HMGD1 and H1, we counted the number of ChIP-seq nucleosome centers within each region, and divided by the total number of mapped reads from each experiment. Compared to total nucleosomes, HMGD1 is depleted in intergenic regions and is highly enriched at promoters. Conversely, H1 is depleted in promoter regions (Figure 2B and Additional file 1: Figure S2). To further understand the HMGD1 promoter enrichment, we split promoters into regions that are downstream and upstream of the TSS. HMGD1 is enriched within both upstream and downstream regions of TSSs, which suggests that it may be associated with both transcriptional initiation (promoter clearance) and elongation.

**Figure 3:**
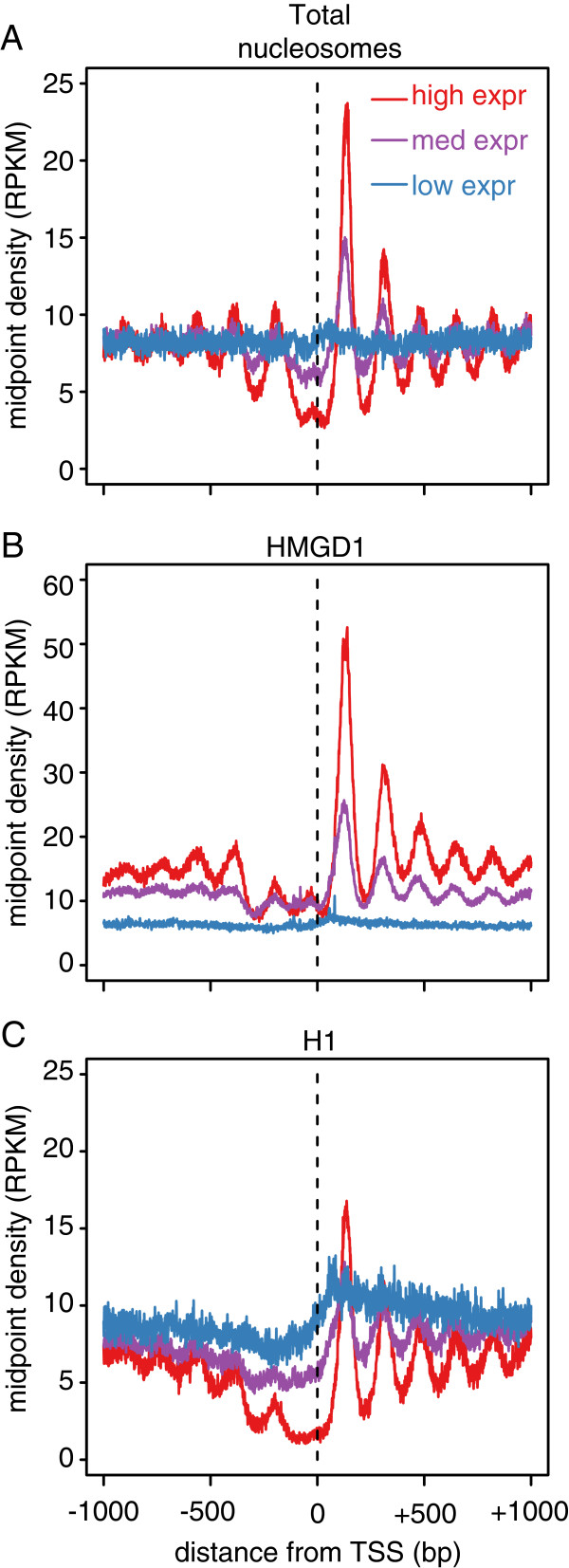
**HMGD1 is enriched around the promoters of active genes and H1 is depleted. (A)** Density of total nucleosome midpoints from S2 cells around transcription start sites (TSSs). Midpoints are aggregated across genes with high, medium and low expression. **(B)** Density of HMGD1 ChIP-seq midpoints around TSSs in S2 cells for genes with high, medium and low expression. **(C)** Density of H1 ChIP-seq midpoints around TSSs in S2 cells for genes with high, medium and low expression.

We next asked whether differences in HMGD1 and H1 binding correlate with transcriptional activity. We utilized RNA-seq data from S2 cells [34] and grouped genes by their expression levels. We then aggregated data across genes in each expression group and compared the density of HMGD1 and Histone H1 to the density of total nucleosomes. Total nucleosome reads from MNase-seq were used to subtract out effects that are solely attributable to nucleosome positioning (Figure 3A). We find that HMGD1 is highly enriched around the promoters of genes with high expression (Figure 3A & B), while in contrast H1 is preferentially enriched in the promoters of genes that are silenced or have low expression (Figure 3A & C). We further validated the presence of HMGD1 and H1 at a subset of promoters with high and low expression genes using ChIP followed by RT-qPCR (Additional file 1: Figure S3A, S3B & S3C).

**Figure 4:**
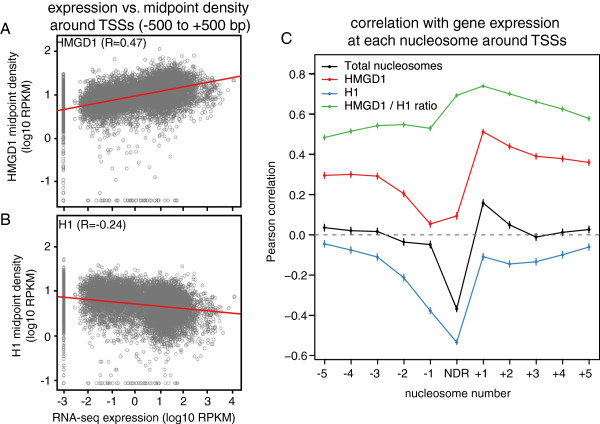
**HMGD1 and H1 are correlated with gene expression. (A)** Scatter plots of normalized HMGD1 and **(B)** H1 versus RNA-seq gene expression. Each data point represents a single gene. The HMGD1 rate is the log2 ratio of HMGD1 midpoints to total nucleosome midpoints from the promoter region -100 to +500 bp. The H1 rate is calculated similarly but for the region -550 to +50 bp. **(C)** Pearson correlations of RNA-seq gene expression with HMGD1 (red), H1 (blue), total nucleosome (black) or the HMGD1/H1 ratio (green). The correlations are computed for non-overlapping genomic regions corresponding roughly to the locations of well-positioned nucleosomes and the nucleosome depleted region (NDR). Vertical line segments represent 95% confidence intervals for the correlations.

To quantify the relationship between gene expression, HMGD1, and H1, we calculated the Pearson correlation between gene expression and ChIP-seq read depth across promoter regions (Figure 4). HMGD1 showed a strong positive correlation (R = 0.71) and H1 had a negative correlation (R = -0.47) with gene expression (Figure 4A & B). We next divided promoters into several non-overlapping regions corresponding to the approximate locations of well-positioned nucleosomes and the nucleosome-depleted region (NDR) (Additional file 1: Figure S2). For each region we then calculated the correlation across all promoters. HMGD1 has a moderately strong positive correlation with gene expression both upstream and downstream of the promoter, with a maximum correlation at the +1 nucleosome (R = 0.50; P < 10^-15^) (Figure 4C). H1 has a negative correlation with gene expression, which is strongest at the nucleosome-depleted region immediately upstream of the TSS (R = -0.55; P < 10^-15^) (Figure 4C). These data suggest that while chromatin binding of HMGD1 is associated with transcription activation, H1 may be involved in gene silencing or repression.

If HMGD1 and H1 bind mutually exclusively and reciprocally to the same sequences, then the ratio of HMGD1 to H1 should be more strongly associated with gene expression than either measure alone. To assess this we computed the correlation between gene expression and the ratio of HMGD1 to H1 density. The correlations obtained with this ratio are substantially and significantly stronger than those with H1 or HMGD1 (P < 10^-15^ for every region by F-test), and reach a maximum of R = 0.73 at the +1 nucleosome. This argues that the relative levels of these proteins are more important than their individual absolute levels (Figure 4C).

### HMGD1 is associated with DNase I hypersensitive sites

**Figure 5:**
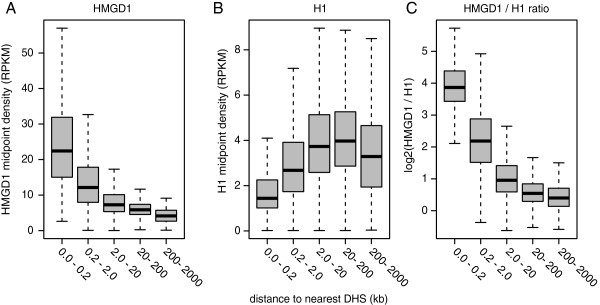
**HMGD1 and H1 density are correlated with distance from DNaseI hypersensitive sites.** Box plots show **(A)** the distribution of HMGD1 and **(B)** H1 densities calculated from non-overlapping 1 kb regions. The box represents the inter-quartile range of the distribution and the bar represents the median. The whiskers extend to the most extreme data point that is no more than 1.5 times the interquartile range from the box. Regions are grouped by their distance to the nearest DNase I hypersensitive site (DHS). **(C)** Distributions of HMGD1/H1 ratio for DHS regions less than 2 kb from the nearest annotated TSS.

HMGN1, the human homolog of HMGD1, is known to co-localize with DNaseI hypersensitive sites [35-37], which are sensitive indicators of open chromatin [38-40]. We wondered whether HMGD1 would be generally associated with open chromatin regions, including those outside of promoter regions. To address this question, we obtained a set of DHSs for *Drosophila* S2 cells [34]. We then divided the *Drosophila* genome into non-overlapping 1 kb regions and computed the distance of each region to the nearest DHS. The density of HMGD1 nucleosome midpoints is much higher near DHSs, and there is a negative correlation between DHS distance and HMGD1 density (R = -0.31; P < 10^-15^) (Figure 5A). H1 density has only a very weak positive correlation with DHS distance (R = 0.091; P < 10^-15^) (Figure 5B).

Collectively our data suggest a high degree of correspondence between HMGD1 and regions of open chromatin in S2 cells. In contrast, chromatin regions containing H1 are less accessible and perhaps refractory to DNase I. Previous studies have argued that HMGD1 and H1 compete for binding to the same linker DNA regions [41]; thus the accessibility of chromatin to regulatory factors could be mediated by competition between these proteins. If chromatin accessibility is driven by competition between these proteins, then their relative abundance should be more predictive than the level of either protein on its own. We tested this prediction by computing the ratio of HMGD1 to H1 with respect to the distance to DHSs (Figure 5C). The correlation with the ratio is significantly stronger than obtained by either protein on its own (P < 10^-15^ for both comparisons by F-test), Even after excluding all regions that are within 2 kb of a known TSS, the HMGD1/H1 ratio is highest near to DHSs (Additional file 1: Figure S4). Our data suggest that the relative levels of HMGD1 to H1 binding may be a primary determinant of chromatin openness and are consistent with previous experimental results that suggest these proteins bind competitively in vivo.

### HMGD1 and H1 genome-wide binding regions align with transcriptionally active and inactive epigenetic marks respectively

**Figure 6:**
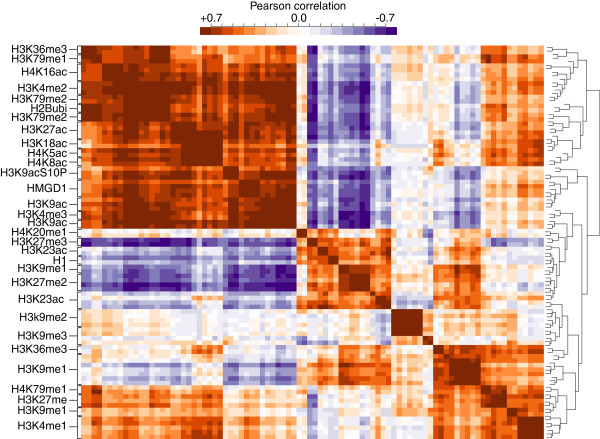
**Hierarchical clustering of HMGD1 and H1 with histone post-translational modifications at TSSs.** The colors in the heat map indicate the pairwise correlation between histone PTM signals from ChIP-chip experiments, H1 density and HMGD1 density. Correlations were computed from 2 kb regions centered on the TSSs of annotated genes. Blue indicates negative correlation, white indicates no correlation and orange to red indicates positive correlation.

Histone posttranslational modifications (PTMs) direct many important processes such as gene activation and repression [42-47]. To determine whether H1 and HMGD1 are associated with specific core histone PTMs, we downloaded a large set of S2 cell line histone modification data from the modENCODE project (Kharchenko et al., [34]). Using 2 kb windows surrounding each annotated TSSs, we computed the mean signal for each PTM and estimated the density of HMGD1- and H1–bound nucleosomes. We then calculated the pairwise correlations across TSS regions for all possible combinations of PTMs, HMGD1 and H1 and performed hierarchical clustering to visualize the results (Figure 6 and Additional file 1: Figure S5). We found that HMGD1 clusters with a large number of activating PTMs including H3K36me3, H3K79me1/2, H3K27ac, H3K18ac, H3K9acS10P, H3K9ac, H3K4me2/3, H4K16ac, and H4K8ac and with elongating marks H3K36me3, H2Bubi [48,49]. In contrast, H1 clusters with several repressive marks, namely H3K27me2/3, H3K9me3, and H4K20me1, which are known to be associated with gene silencing and heterochromatin.

### HMGD1 and H1 are associated with local nucleosome spacing

**Figure 7:**
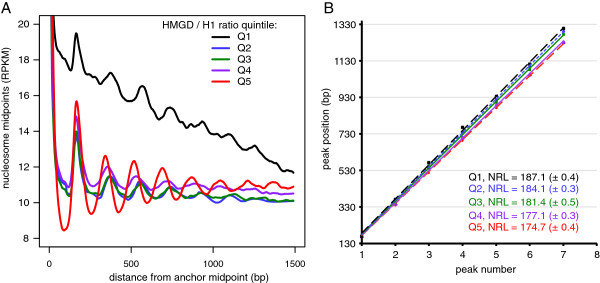
**Nucleosome repeat length (NRL) is associated with HMGD1/H1 ratio. (A)** Density of total nucleosome MNase-seq midpoints as a function of distance from an anchor midpoint. The genome was divided into non-overlapping 1 kb regions from which anchor midpoints were taken. Regions were stratified by HMGD/H1 quintile, as indicated by the colored lines. **(B)** Plot of the peak position versus the peak number. The NRL for each HMGD/H1 quintile is estimated from the slope of a line fit by least-squares.

The average distance between two nucleosomes, the nucleosome repeat length (NRL), is an important parameter that describes primary chromatin organization. Since H1’s expression level is a key determinant of the genome-wide NRL [10,25], we sought to determine whether the relative protein levels of HMGD1 and H1 are associated with local differences in nucleosome spacing. We divided the genome into non-overlapping 2 kb regions and computed the ratio of HMGD1 to H1 in each region. We then calculated the density of nucleosome midpoints as a function of distance from an “anchor” nucleosome (treating each nucleosome midpoint within a region as an anchor in turn). The resulting plots show well-defined peaks in nucleosome occupancy particularly for regions with the highest HMGD1/H1 ratio (Figure 7A). These peaks were then used to estimate the NRL, which we found to be better correlated with the ratio of HMGD1 to H1 (Figure 7B) than either H1 or HMGD1 alone, (Additional file 1: Figure S6A and S6B). The NRL varies from 174 bp in regions with a high HMGD1/H1 ratio to 187 bp in regions with a low HMGD1/H1 ratio. These results suggest that the stoichiometry of H1 is important, not only for the average NRL across the genome, but also for defining the local spacing of nucleosomes. Potentially, the spacing between nucleosomes may be specified by which CAP is bound to the linker region (e.g. HMGD1 or H1). Under this hypothesis the average NRL across cells would reflect the relative concentrations of these proteins.

### H1 and HMGD1 bind reciprocally and affect gene expression and nucleosome repeat length

**Figure 8:**
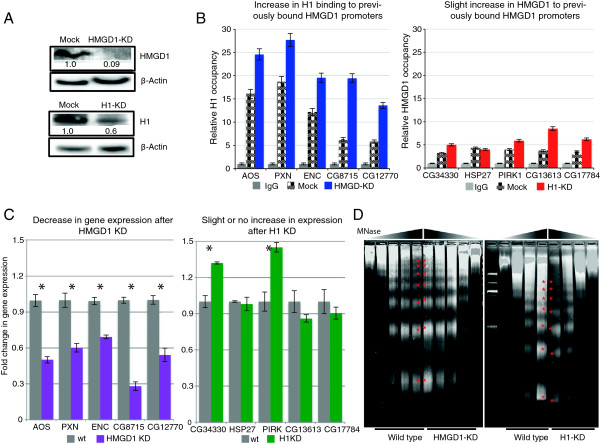
**HMGD1 and H1 knockdown reciprocally mediate higher order chromatin structure leading to changes in distinct gene expression outcomes. (A)** S2 cells were treated with the indicated siRNA for 48 hours and knockdown (KD) was validated by western blot. For the H1 KD, there was a ~40% reduction in H1 protein levels; for the HMGD1 KD, there was a ~90% reduction in HMGD1 protein levels. **(B)** Relative occupancy of HMGD1 and H1 as measured by ChIP-qPCR at promoters that were initially bound by H1 and HMGD1. Relative occupancy was computed by setting the IgG control to 1. **(C)** Gene expression changes measured by RT-qPCR following HMGD1 or H1 knockdown in S2 cells. Expression was normalized to β-actin levels and fold change in expression was calculated by setting the expression in the mock control to 1. In **(B)** and **(C)**, error bars are mean ± SD from three independent experiments. Using a student t-test, the p values from all experiments were significant with values ranging from p = 1.35 × 10^-4^ to p = 2.87 × 10^-8^**(D)** Nucleosome repeat length changes caused by KD of H1 or HMGD1. S2 nuclei (5 × 10^6^) were digested with 25 units of MNase for 1, 3, 5, 7 and 10mins. Purified DNA from these digests was run on a 3.3% Nusieve™ agarose gel. M indicates DNA ladders. Red stars indicate the nucleosome ladders. A decrease in H1 levels decreased the nucleosome repeat length by ~24 bp. Knockdown of HMGD1 increased the nucleosome repeat length by ~7 bp.

To directly test the hypothesis that reciprocal binding of HMGD1 and H1 has functional consequences for gene regulation and nucleosome spacing, we performed siRNA knockdown of both HMGD1 and H1 (Figure 8A) [31,50]. Following both knockdowns, we assayed the occupancy of H1 and HMGD1 in representative promoters using ChIP-qPCR (Figure 8B and Additional file 1: Figure S3). For the HMGD1 knockdown, the subset of promoters with high initial levels of HMGD1 showed a remarkable two- to three-fold increase in H1 binding and a significant reduction in gene expression (Figure 8B and C). Conversely, H1 knockdown resulted in only modest HMGD1 promoter occupancy increases at 4 of the 5 target H1 promoters examined and no change was observed at one promoter, HSP27. Likewise, observed changes in gene expression were varied at these promoters with only two genes showing significant increases in expression (Figure 8B and C). This suggests that H1 depletion alone may not be sufficient to initiate gene expression in all heterochromatic regions. On the other hand, the reduction in gene expression following HMGD1 knockdown suggests that HMGD1 facilitates transcriptional initiation or elongation.

Interestingly, knockdown of H1 decreased the average NRL in bulk chromatin by ~24 bp, whereas HMGD1 knockdown increased the average NRL by ~7 bp (Figure 8D and Additional file 1: Figure S7). H1 knockdown may have a greater effect on the repeat length of bulk chromatin because it is present in most regions of the genome, while HMGD1 is most abundant at highly transcribed regions that comprise a smaller fraction of the genome. These results suggest that nucleosome spacing may be dictated by the binding of these (and potentially other) CAPs.

## Discussion

The role of the core histones and their modifications in gene regulation has been widely studied, however, the function of chromatin architectural proteins such as histone H1 and the high mobility group proteins (HMGs) are less well understood. In this study we used genomic, gene specific and biochemical approaches to characterize two highly abundant chromatin architectural proteins: H1 and HMGD1. Additionally, by using a homogenous cell population and high-throughput sequencing, we were able to directly correlate H1 and HMGD1 protein binding with gene activity and chromatin structure on a genome-wide scale. We found that HMGD1 is associated with active transcription and euchromatin, while H1 is associated with repressed genes and heterochromatin. More specifically, HMGD1 occupancy is highest in regions with active transcription, activating histone PTMs, and DHSs, while H1 is enriched in inaccessible regions with low gene expression, repressive histone PTMs and low DNase I sensitivity. Our results for HMGD1 are consistent with those from recent studies of HMGN1 (the human homolog of *D. melanogaster* HMGD1), which found that this protein is enriched around DNaseI hypersensitive sites at the promoters of actively transcribed genes [35]. This suggests that the HMGD1 distribution at promoter regions is likely a broadly conserved feature of HMG proteins. Recently, genome-wide distributions of the histone H1 variants H1d and H1c have been obtained from human embryonic stem cells [51]. As with histone H1 in Drosophila S2 cells, these histone variants are depleted from regions of active chromatin and are enriched in heterochromatic regions [51].

Our results suggest that H1 and HMGD1 are part of a mechanism that establishes or maintains repressed and active chromatin states. How HMGD1 or H1 are recruited to distinct chromatin regions remains unknown, but our data argue that reciprocal binding of these architectural proteins to the same chromatin region is important [22]. The high on/off rates of these proteins’ chromatin association may provide a window of opportunity where one CAP can replace the other to cause a change in chromatin state. For instance, specific HMGs may be able to displace H1 and locally destabilize chromatin so that other proteins can be recruited to initiate transcriptional activation [52-54]. Consistent with this notion, we found that depletion of HMGD1 results in H1 binding to previously HMGD1-bound promoters (Figure 8B), with a subsequent decrease in gene expression. On the other hand, depletion of H1 only slightly increases in HMGD1 occupancy at a subset of previously bound H1 promoters, and had varied effects on gene expression, with 2 of 5 genes showing a significant increase in expression (Figure 8).

The expression level of H1 was previously known to affect the genome-wide average NRL [25]. We found additionally that depletion of HMGD1 affects the genome-wide NRL. We hypothesize that HMGs may be responsible for the reduction in nucleosome repeat lengths in active chromatin regions [55]. Changes in NRL are likely very important in gene regulation, because even a single base pair (bp) shift in linker length changes the DNA helical twist, resulting in a 36° torsional angle change in the neighboring nucleosome position [56]. It is therefore possible that changes in nucleosome spacing introduced by CAPs such as HMGD1 modulate the accessibility of regulatory factor binding sites and have downstream consequences on transcription factor binding and gene expression.

## Conclusion

In summary, we have shown that HMGD1 is localized to genomic loci with transcriptionally active histone modifications and DNase I hypersensitive sites, whereas H1 is primarily associated with heterochromatic regions. Both proteins contribute to the spacing of nucleosomes and it is likely that the depletion of H1 in transcriptionally active regions reflects the competitive and mutually exclusive binding of HMGs to linker sequences. While we have studied the genomic localization of H1 and HMGD1, other CAPs are also likely to play important roles in gene regulation, nucleosome spacing and chromatin compaction. For example, competition between H1 and Poly-ADP-ribose polymerase can elicit specific transcriptional outcomes [54]. An important future direction will be to study how CAPs compete with each other and whether their different isoforms and post-translational modifications affect their function. Already some data suggest that H1 and HMGD1 act differently depending on their post-translational modifications or binding partners [28,30,53,57-60]. Finally with this data, we provide a platform to determine in fine detail, the interplay between chromatin architectural proteins, epigenetic factors (histone PTMs, PTMs on CAPs themselves, DNA methylation) and gene expression.

## Methods

### S2 cell culture and siRNA knockdown

*D. Melanogaster* S2-DRSC cells (obtained from the *Drosophila* Genomics Resource Center) were cultured in Schneider’s *Drosophila* medium (Invitrogen) supplemented with 10% FCS (Hyclone). Knockdowns in S2 cells were done using the following constructs: an H1 construct obtained from [11] and HMGD1 PCR products were obtained from the Drosophila RNAi screening center (DSRC). Production of both dsRNA and transfection were done according to protocols from DSRC.

### Antibodies

The following antibodies were used in this study: anti-H1 (Active motif- 39575), anti-HMGD1 (custom-made antibody from Thermo Fisher Scientific), anti-H4 (Abcam-ab10158), anti-H4K16ac (Activ motif 39167).

### Salt fractionation of chromatin

S2 cell chromatin was fractionated into putative euchromatin, heterochromatin, and pelleted heterochromatin using a modification of the method of [61]. S2 cells were incubated with NP-40 lysis buffer (10 mM Tris [pH 7.4], 10 mM NaCl, 3 mM MgCl_2_, 0.5% NP-40, 0.15 mM spermine, 0.5 mM spermidine, complete Protease Inhibitor Cocktail). After two washes with wash buffer A (10 mM Tris [pH 7.4], 15 mM NaCl, 60 mM KCl, 0.15 mM spermine, 0.5 mM spermidine), nuclei were resuspended in ice-cold MNase digestion buffer (10 mM Tris [pH 7.4], 15 mM NaCl, 60 mM KCl, 0.15 mM spermine, 0.5 mM spermidine, 1 mM CaCl_2_). Chromatin was digested with predetermined MNase concentration at RT for 5 mins. The digestion reaction was stopped by the addition of EDTA to a final EDTA concentration of 10 mM (on ice). Undigested chromatin (P1) was removed by centrifugation at 14,000 × g for 30 min at 4°C. 200 mM MgCl2 was added dropwise (end concentration = 2 mM), to the supernatant while stirring at 4°C. After a 4°C overnight rotation, the suspension was centrifuged as above resulting in a magnesium insoluble DNA-pellet (P2) and magnesium soluble-supernatant (S2) fraction (Pellet in Figure 1C).

### Nucleosome repeat length (NRL) determination in knockdown cells

30 × 10^6^ cells were used for each type of experiment. From these, nuclei from wildtype and knockdown cells were extracted and MNase digestion was performed as described above (Salt fractionation of chromatin). Digestion was done at different time points at room temperature with predetermined MNase concentration. DNA was purified and separated on a 3.3% Nusieve agarose gel. The NRL at each time point was calculated using the regression line generated with size (bp) of polynucleosomes [25,62], and the values extrapolated to time “0” as previously described [63].

### Sucrose gradient fractionation of chromatin

S2 cells were crosslinked with 1 ml of 1% formaldehyde in PBS for 10 min at room temperature to preserve chromatin structure. The protocol was performed according to [33] with minor modifications. The crosslinked reaction was stopped by the addition of 0.125 mM glycine and cells were washed with ice-chilled PBS twice. Next cells were washed with NP-40 lysis buffer (10 mM Tris [pH 7.4], 10 mM NaCl, 3 mM MgCl_2_, 0.5% NP-40, 0.15 mM spermine, 0.5 mM spermidine, complete Protease Inhibitor Cocktail). After two washes with wash buffer A (10 mM Tris [pH 7.4], 15 mM NaCl, 60 mM KCl, 0.15 mM spermine, 0.5 mM spermidine), nuclei were resuspended in ice-cold MNase digestion buffer (10 mM Tris [pH 7.4], 15 mM NaCl, 60 mM KCl, 0.15 mM spermine, 0.5 mM spermidine, 1 mM CaCl_2_). Chromatin was digested with a predetermined MNase concentration at RT for 5 min to yield a wide MW size range of the DNA (0.1 to > 2 kb). The lysates were then spun down briefly (700 *g*, for 5 min) to remove debris, and layered onto a 9.5 ml sucrose gradient (6–40%) in 1.1% Triton X-100, 0.01% SDS, 16.7 mM Tris–HCl (pH 8.0), 1.2 mM EDTA, 167 mM NaCl, and complete Protease Inhibitor Cocktail (sigma P8340) in a polyallomer centrifuge tube (#331374, Beckman). Ultracentrifugation was run at 43,000 × g for 3 h at 4°C. 0.5-ml and fractions were collected from the gradient by pipetting from top to bottom using a micropipette. Aliquots from each fraction were analyzed for protein or DNA analyses. For DNA analyses, aliquots were reverse crosslinked at 65°C overnight, treated with RNase A and Proteinase K (Sigma R4642 and P4850 respectively), followed by extraction with phenol/chloroform for DNA analyses. After precipitation by ethanol supplemented with 10 μg of glycogen, purified DNA from each fraction was loaded onto a 3% nusieve agarose gel in a Tris–glycine buffer. Protein samples were run on 8 - 12% SDS Invitrogen NuPAGE gels (part # NP0322BOX) and analyzed further by western blot.

### ChIP of H1 and HMGD1 bound nucleosomes

Chromatin fixation and immunoprecipitation were performed essentially as described by [64,65] with minor modifications. Cells (7.5 × 10^8^) were fixed in 10 mL of medium with 1% formaldehyde for 10 min at room temperature. Cross-linked cells were digested with micrococcal nuclease (Sigma N3755) to produce chromatin fragments of an average size of ~450 bp equivalent to ~2-3 nucleosomes. Soluble chromatin was separated from insoluble material by centrifugation. The supernatant containing chromatin was used for immunoprecipitation. Resultant eluates were subjected to both agarose gel electrophoresis for DNA analyses and western blots for protein analyses. To determine whether formaldehyde crosslinking and pull-down with respective antibodies allowed efficient pull-down of H1- or HMGD1-bound nucleosomes, we probed eluates with antibodies for our proteins of interest and H4. No mononucleosomal DNA or histone H4 enrichment was observed in the IgG controls, suggesting that pull downs were sufficiently selective. Finally, the mononucleosomal fragments from these ChIP experiments were adapted for deep-sequencing analyses via paired-end or single-end SoLiD life technology and sequenced to high coverage. In total, we created 2–4 replica each for each ChIP.

### Immunoprecipitation-quantitative real-time PCR assay

ChIP was performed on MNase digested chromatin fragments like before using anti-H1 and anti-HMGD antibodies. 6 μg of antibody, 100 μl of dynal beads and 400 μg of chromatin DNA were used for each ChIP. IgG was used as a no-antibody control. Eluted DNA was subjected to real-time PCR using the PerfeCTa SYBR Green FastMix (Quanta Biosciences Inc, USA) and Biorad CF96 following the manufacturer’s instructions.

The primers used to amplify selected gene promoters were as follows:

CG13613:

F: 5’-AGACAATGAAGTGGTCTGGATT-3’; R: 5’ATCAGGGTGATCAGCAGCAG-3’;

CG17784:

F: 5’-TCGTGGCTGAGATCCAAGTTT-3’; R: 5’-CAGGTCAGAACTCTGTGGACC-3’;

CG8715:

F: 5’-ACTCAGAATCCAGTCAGCACAG–3’; R: 5’-CGCCTGAACGAGTTTGTGTG–3’;

CG12770:

F: 5’-GGCCACACTGTCAAATCCCT-3’; R: 5’-AGCTCGGGACTTTGTTCCTG-3’

20 μl reactions were set up using 1 μl of ChIP DNA, 10 μl of 2× PerfeCTa SYBR Green FastMix (Quanta) and 0.5 μM each of gene specific primers. The cycling conditions were: 95°C for 2:00 m; 40 cycles of 95°C for 30 s, 55°C for 30 s and 72°C for 1:00 m. Fluorescence was measured right after each elongation step. Dissociation curves were used to confirm specificity of PCR products. The “signal over background” normalization method was used to calculate fold enrichment from Ct values.

### Gene expression and real time quantitative PCR

Total RNA was isolated using QIAGEN RNeasy according to the manufacturer’s extraction protocol (Qiagen 74106) which included the DNase step (Qiagen 79254). cDNA was generated from 1 μg of total RNA using the Superscript III First-Strand Synthesis System (Life Technologies). Analysis of mRNA was then accomplished using primers specific to each of the target mRNAs. RT-qPCR reactions were performed using PerfeCTa SYBR Green FastMix (Quanta Biosciences Inc, USA) and Biorad CF96 following the manufacturer’s instructions and the resulting Ct values were normalized to ||-actin. Primers used are listed below:

CG13613:

F: 5’-CTCCAGCTGACCTCATCCAT-3’; R: 5’-TTCATCTGGAAGCCCATGTC-3’

CG17784:

F: 5’-CAGACCGACAAGGAGCAGTC-3’; R: 5’-GTGTTCCAAAAGCTCCACCA-3’

CG8715:

F: 5’-ACGTGTTAAGCTGCCACCAC-3’; R: 5’-CACGTCCAGGTAGCCAATGT-3’

CG12770:

F: 5’-AAGCTACGCCTGCAGATCAA-3’; R: 5’-GGACAGGCGATTCATGTTGT-3’

### Preparation of ChIP-seq libraries

Sequencing libraries were prepared with the SoLiD ABI technologies as described in [66]. After adapter ligation, library fragments of ~160 bp were isolated from agarose gel. The DNA was PCR amplified with SoLiD primers for 10 cycles, purified, and loaded on a SoLiD flow cell for cluster generation.

### Gene sets

The gene sets used in these analyses were from BDGP Release 5 and the Release 5.12 annotations (Oct. 2008) provided by FlyBase.

### MNase-seq and Nucleosome-ChIP-seq processing

HMGD1 colorspace fasta reads were converted to fastq format by the University of Chicago Genomics core using a custom script. We sorted HMGD1 reads into separate library files based on their barcodes, and mapped them to the Drosophila genome using BWA v0.6.1 [67]. We estimated the mean fragment length of each library by computing the offset that gave the highest covariation of read depth between the forward and reverse strands. We estimated HMGD1 nucleosome midpoint locations as the read start plus the offset (or minus the offset for those that mapped to the reverse strand).

We aligned the MNase-seq and H1 paired-end reads to the genome using the standard SOLiD pipeline [68] and discarded those where only one side of pair mapped. We estimated the distribution of fragment sizes from separation of read pairs, and discarded read pairs outside of the central 95% of distribution (101-191 bp). We estimated nucleosome midpoints as the midpoint between read pairs.

### Gene expression

We downloaded aligned RNA-seq reads from S2 cells in BAM format from modENCODE [69]. We counted the number of sequence tags that overlapped with annotated exons in each transcript, added a pseudocount and normalized by transcript length to obtain reads per kb per million mapped reads (RPKM). For most analyses we used the log RPKM value as our expression measurement.

### Correlations with gene expression

We divided regions around each TSS into windows that corresponded to the typical locations of each positioned nucleosome and the nucleosome depleted regions as estimated from aggregate gene plots (Figure 3). We computed the Pearson correlation between gene expression and the density of H1, HMGD, and nucleosome midpoints (center) density in each region. We additionally computed correlation with the ratio log2 (HMGD density/H1 density). To assess whether the ratio gave a significantly better fit that either measure alone, we compared F-statistics from the model expr ~ log2 (H1) + log2 (HMGD) to those from the univariate models expr ~ log2 (H1) and expr ~ log2 (HMGD).

### Correlations with DHS distance

We obtained a list of previously identified DHSs for S2 cells [34] and calculated the distance to the nearest DHS for each base in the genome. We divided the genome into non-overlapping 1 kb windows and assigned each window the following values: *DHS_dist* = *log2(mean(DHS_distance))*, *HMGD1* = *log2(mean(HMGD1_midpoint_depth))* and *H1* = *log(mean(H1_midpoint_depth))*. We then calculated Pearson correlations across windows and fit linear models by least squares. We used an F-test to compare the fit of the linear model *DHS_dist* ~ *HMGD1* + *H1* to that of the models with only one predictor (*DHS_dist* ~ *HMGD1* and *DHS_dist* ~ *H1)*. The linear model with both predictors gave a significantly better fit than the single predictor models (P < 10^-15^ for both).

### Correlations with histone modifications

We downloaded processed and smoothed ChIP-chip data from the modENCODE project [34]. We used data from a total of 84 experiments (including replicates) that were performed in *Drosophila* S2 cells using antibodies for specific histone post-translational modifications. We took 2 kb windows surrounding all annotated TSSs and computed mean values for each histone modification experiment in each window. For the same windows, we also computed normalized values for the H1 and HMGD1 experiments by dividing the number of midpoints from a given experiment by the number total nucleosome midpoints and taking the log. We then computed Pearson correlations, *R*, across windows for all possible pairs of experiments and performed hierarchical clustering of the experiments, using 1-*R* as a distance metric.

### Data access

All data are publically available from GEO (http://www.ncbi.nlm.nih.gov/geo/) under accession GSE49526.

## Abbreviations

TSS: Transcription start sites; PTMs: Post translational modifications; ChIP: Chromatin immunoprecipitation; nuc-ChIP-seq: Nucleosome immunoprecipitation followed by next generation sequencing; MNase: Micrococcal nuclease; RT-PCR: Real-time polymerase chain reaction.

## Competing interests

The authors declare that they have no competing interests.

## Authors’ contributions

NN, RM, JM, RM, and YNF-M performed the experiments. NN, and YNF-M performed the experimental analyses. GM, JKP and YNF-M performed the bioinformatics analyses. NN, GM, RM, JM, JKP and YNF-M wrote the paper. NN, GM, JKP and YNF-M conceived and designed experiments. YNF-M directed the research. All authors read and approved the final manuscript.

## Supplementary Material

Additional file 1 **Supplementary Information.** This file contains **Figures S1-S7** and **Table S1**. Click here for file
