# Supplementary material for: The chromatin architectural proteins HMGD1 and H1 bind reciprocally and have opposite effects on chromatin structure and gene regulation
Source: BMC Genomics. 2014 Feb 1;15:92. doi: 10.1186/1471-2164-15-92 (PMC3928079; doi:10.1186/1471-2164-15-92)
Supplement: Additional file 1 — Supplementary Information. This file contains Figures S1-S7 and Table S1. [file 1471-2164-15-92-S1.pdf]

Supplemental Figure S1

A.

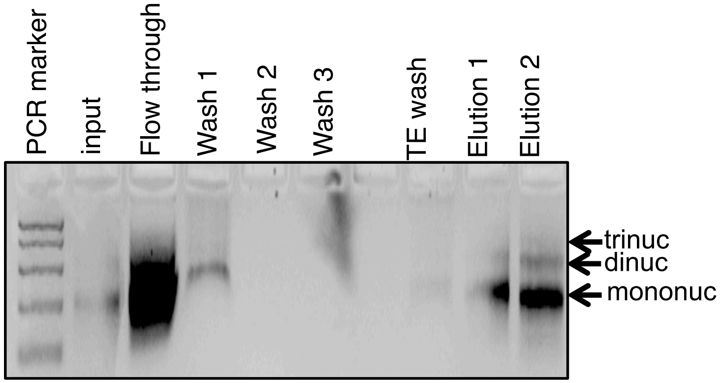

B.

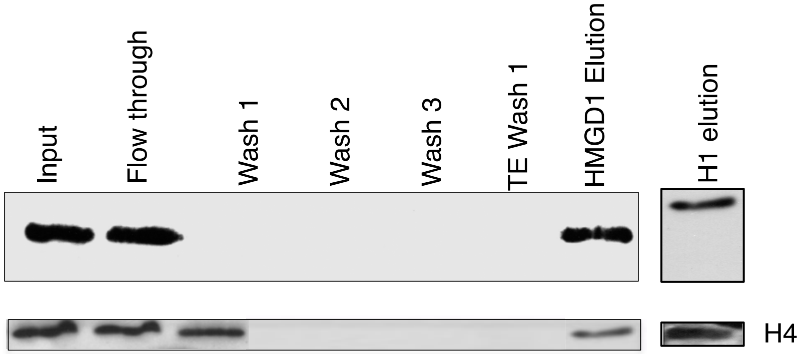

Supplemental Figure S1: ChIP assay. Nuclei from S2 cells were formaldehyde-fixed and chromatin MNase digested to yield ~450bp fragments. Chromatin was subjected to ChIP using antibodies indicated. ***(A)*** Resultant DNA fragments from imput, flow through, several wash steps and elution steps were purified and analyzed on 3% Nusieve™ agarose gel electrophoresis. DNA mononucleosomal fragment (arrow) from elution step was excised and subjected to ABI SoLiD sequencing. Shown is an example of H1-ChIP. In H1 or HMGD1 experiments, mononucleosomal DNA was recovered after elution while no DNA was recovered from the IgG elution. ***(B)*** Proteins from the ChIP experiments were subjected to western blot analyses. Indicated are western blots from H1 and HMGD1 ChIP experiments, H4 antibody is used to indicate samples containing chromatin fractions.

Supplemental Figure S2

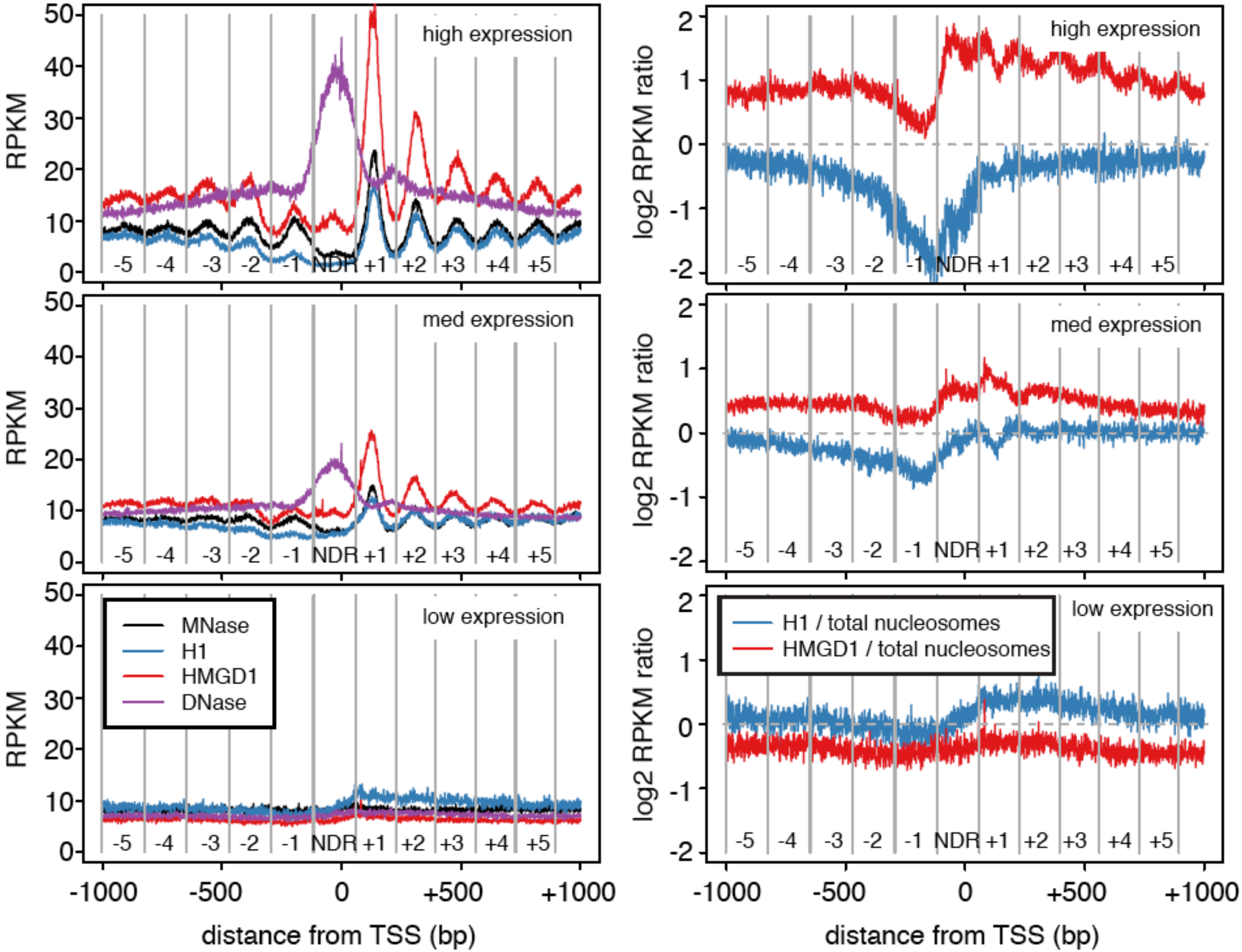

Supplemental Figure S2: HMGD1 and H1 are enriched differently at nucleosomes surrounding transcription start sites. The left panels show midpoints of HMGD1, H1, DNaseI and total nucleosomes aggregated across TSSs. TSSs are grouped into high (upper quartile), medium (two central quartiles) and low expression (bottom quartile). Midpoints are reported in reads per kilobase per million mapped reads (RPKM). The right panels show HMGD1 and H1 normalized by the density of total nucleosomes (log2 RPKM ratios). This emphasizes the enrichment or depletion of HMGD1 or H1 by removing effects that are primarily due to nucleosome positioning. The labels -1, +1, +2, etc. indicate the typical positions of nucleosomes flanking transcription start sites. NDR indicates the nucleosome depleted region.

Supplemental Figure S3

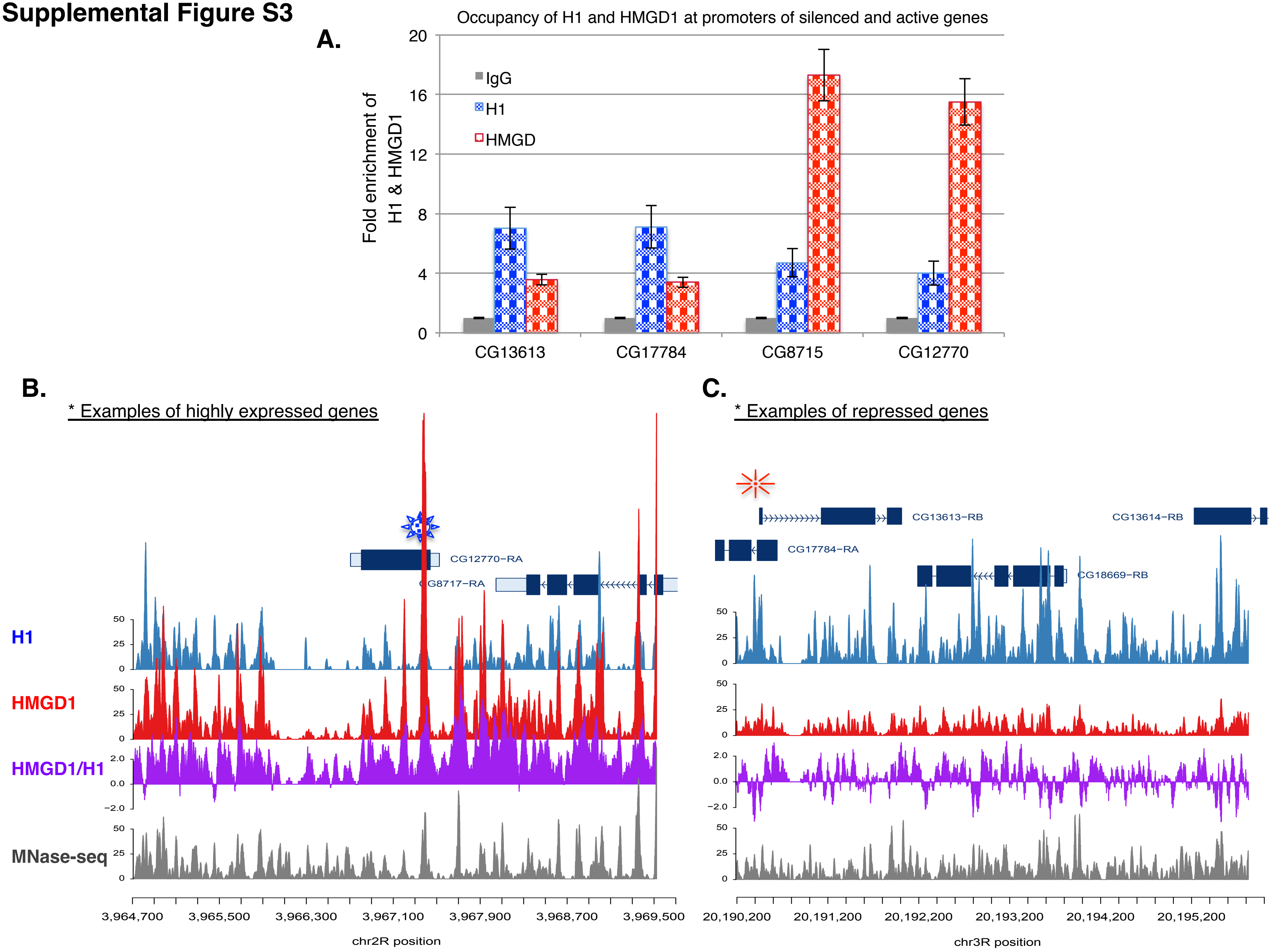

Supplemental Figure S3: Validation by ChIP-qPCR of the enrichment of highly expressed and lowly expressed gene promoters by HMGD1 and H1 respectively. Two panels are shown, exemplifying different promoter regions bound either by HMGD1 (not H1) in B or bound by H1 (not HMGD1) in C. ***(A)*** Validation of HMGD1 and H1 at representative promoters using ChIP-PCR. ***(B)*** HMGD1 binding is significantly enriched at the promoter of CG8715 and CG12770 genes, both of which are expressed in S2 cells. ***(C)*** H1 binds highly to promoters of lowly expressed genes (not HMGD1) such as CG13613 and CG17784. The blue and red graphs represent the amount of nucleosomes mapped from H1-ChIP-seq and HMGD1-ChIP-seq data within the specified genomic region respectively. Stars represent promoters that were analyzed by qPCR. While the purple graph represents the log2 ratio of HMGD1 to H1 nucleosomal reads. Peaks in the purple curve indicate regions with high HMGD1 relative to H1 and troughs show high H1relative to HMGD1. The gray curve represents the total nucleosomes from S2 MNase-seq analyses.

Supplemental Figure S4

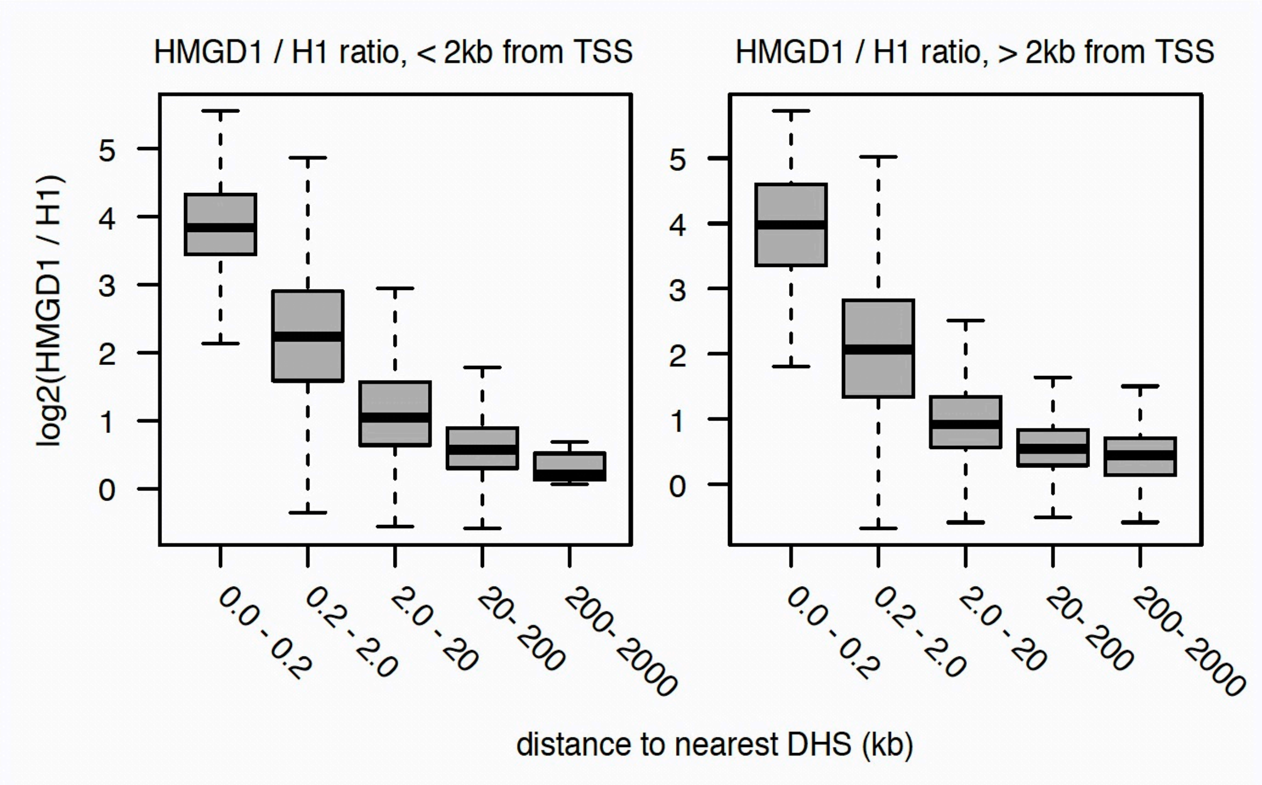

Supplemental Figure S4: HMGD1/H1 ratio is correlated with distance from DNaseI hypersensitive sites to TSS. Box plots show ***(A)*** the distribution of ratio of HMGD1 to H1 at DHS regions less than 2kb from the nearest annotated TSS and ***(B)*** Distributions of HMGD1/H1 ratio for DHS regions that are > 2kb away from an annotated TSS.

Supplemental Figure S5

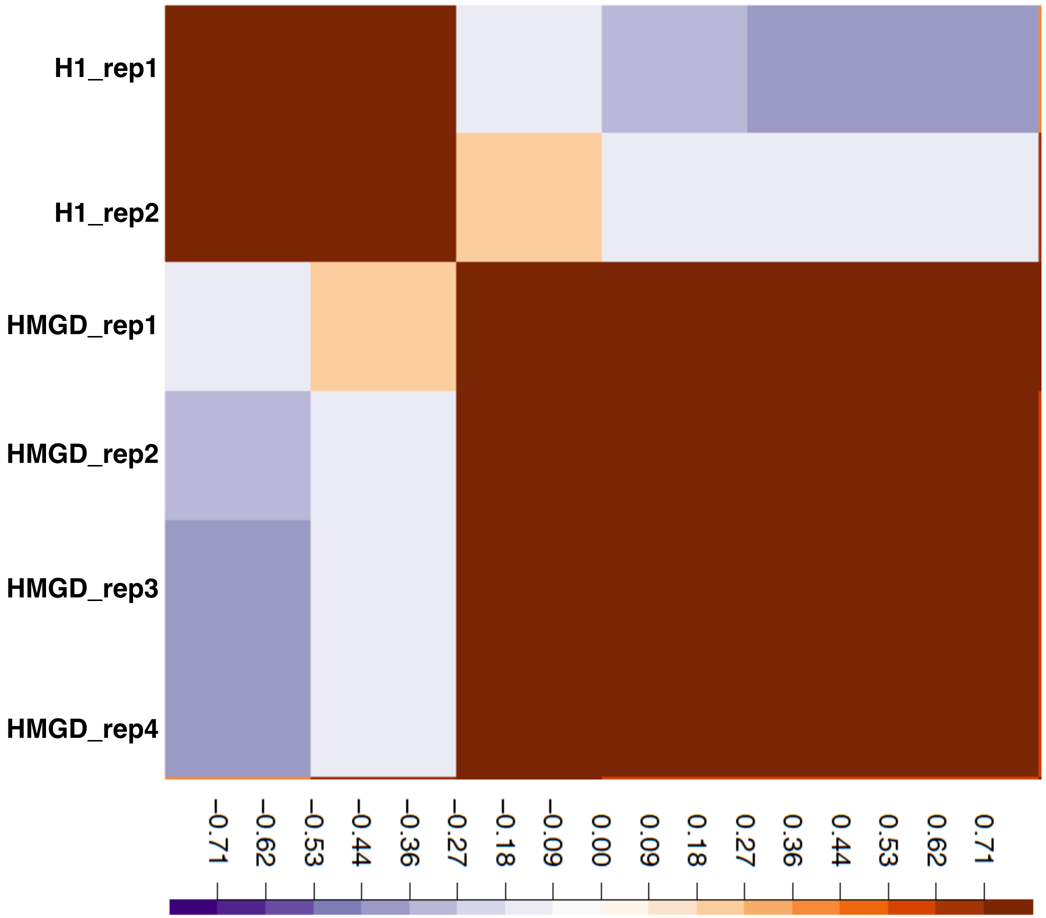

Supplemental Figure S5: Each ChIP-seq data was highly reproducible with each other and correlated to the respective PTMs.

Supplemental Figure S6

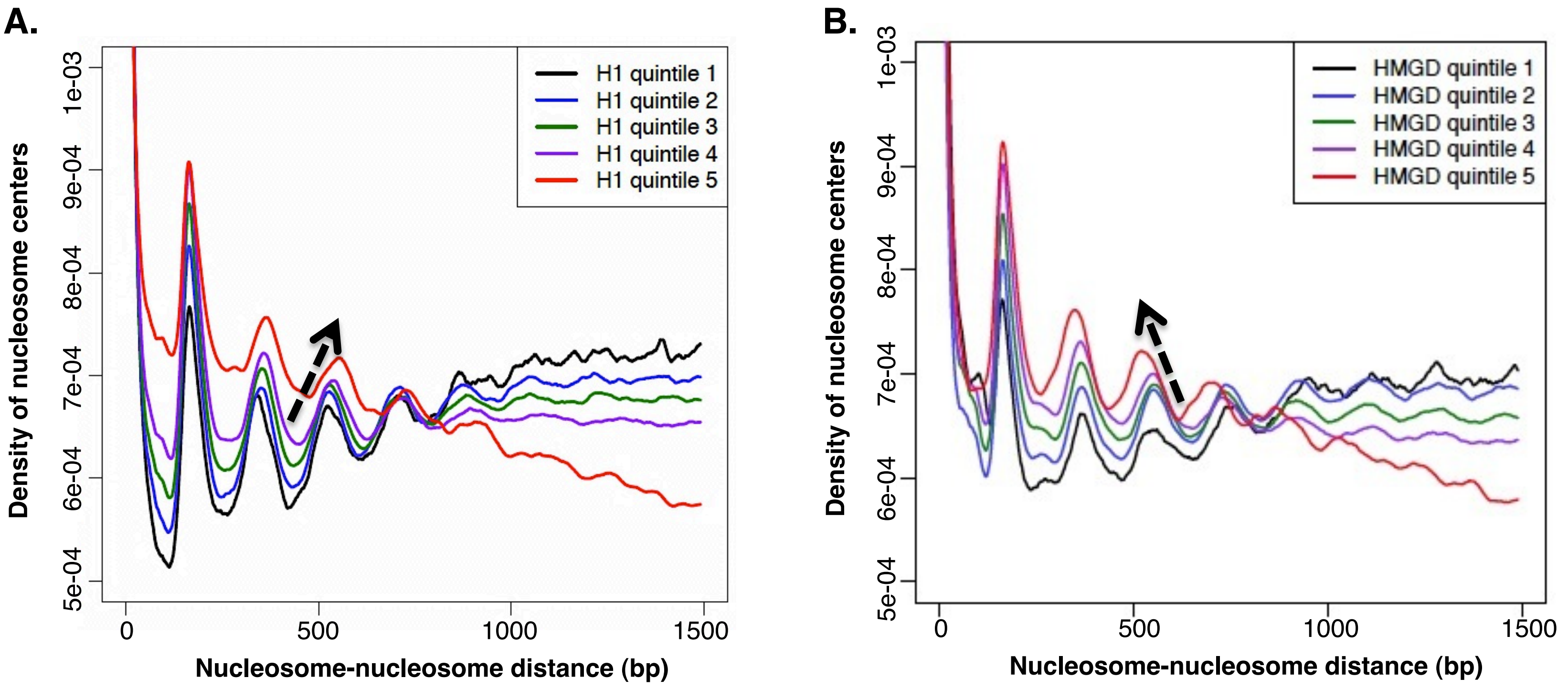

Supplemental Fig. S6: Nucleosome repeat length in chromatin bound by HMGD1 and H1 separated as quintiles of the enrichment of either HMGD1 or H1. ***(A)*** Histogram of distances between nearest neighbor nucleosomes in in H1 nucleosome-ChIP-seq data and ***(B)*** HMGD1 nucleosome-ChIP-seq data Peaks reflect the nucleosome centers. Arrow indicates that as the quintile increases (enrichment of protein binding), so too does the direction of the NRL, either increase as in the case with H1 or decrease with HMGD1 binding.

Supplemental Figure S7

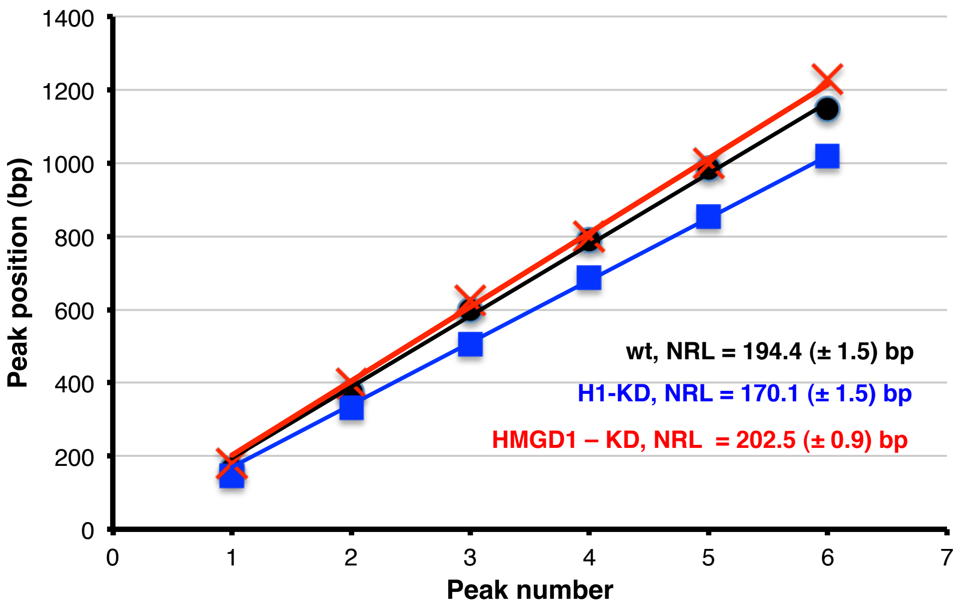

Supplemental Figure S7: Depletion of HMGD1 causes an increase in NRL of ~7bp, while H1 depletion results in ~24bp NRL repeat length decrease.

Supplemental Table 1

# Nucleosomes in Drosophila genome	H1 nucleosome seq data: # Unique pairs of reads	H1 nucleosome seq data: Average # of reads per nucleosome	HMGD1 nucleosome seq data: # Unique pairs of reads	HMGD1 nucleosome seq data: Average # of reads per nucleosome
~613270	11338899	**18**	13533456	**22**

Chromosome Region	DNA Length (bp)	H1: Unique Paired Reads	H1: Number of Nucleosomes	H1: Number of Reads per H1-nucleosome	H1: Normalized number of Reads per Nucleosome	HMGD1: Unique paired reads	HMGD1: Number of Nucleosomes	HMGD1: Number of Reads per HMGD1-nucleosome
**chr 2L**	23011544	1662596	115058	**20**	**24**	2628872	**115058**	**23**
**chr 2LHet**	368872	42758	1844	**7**	**11**	10793	**1844**	**6**
**chr 2R**	21146708	1579276	105734	**20**	**24**	2679198	**105734**	**25**
**chr 2RHet**	3288761	298958	16444	**9**	**13**	106295	**16444**	**6**
**chr 3L**	24543557	1833863	122718	**19**	**23**	2825794	**122718**	**23**
**chr 3Lhet**	2555491	220928	12777	**10**	**14**	85661	**12777**	**7**
**chr 3R**	27905053	2183126	139525	**21**	**25**	3360957	**139525**	**24**
**chr 3RHet**	2517507	316157	12588	**9**	**13**	83227	**12588**	**7**
**chr 4**	1351857	55480	6759	**10**	**14**	98451	**6759**	**15**
**chr X**	22422827	726514	112114	**9**	**13**	1643454	**112114**	**15**
**chr Xhet**	204112	81535	1021	**7**	**11**	7104	**1021**	**7**
**chr Yhet**	347308	144	1737	**0**	**0**	149	**1737**	**0**

Supplemental Table 1: H1-bound nucleosomes are highly enriched at heterochromatic chromosomes as shown by the coverage per nucleosome [determined by the number of reads recovered from each ChIP-seq experiment]. From each experiment, unique reads and only those that mapped to the fly genome were retained. The numbers of reads from each H1- or HMGD1-ChIP-seq experiment were added together to get the total number of reads per experiment type. The mean number of reads from HMGD1 data is ~13.53 X 106 and for H1, ~ 11.34 X 106. In order to calculate the number of reads per nucleosome, we assumed that each chromatosome [nucleosome + linker DNA], covers about ~200bp. Thus, if all the Drosophila genome of ~1.22 X 108bp were covered by a chromatosome, the expected number of nucleosomes is ~6.1 X 104. From this rationale, to calculate how many nucleosome reads were covered by our resultant sequencing data, the number of unique reads were divided against the expected nucleosome number. These results gave ~18 reads per nucleosome in H1 and ~22 nucleosomes per read in HMGD1-ChIPseq data. Similar type of analyses was done to calculate the coverage per nucleosome per chromosome type.
